# Correction to: Changing patterns and clinical outcomes of hospitalized patients with COVID‑19 severe pneumonia treated with remdesivir according to vaccination status: results from a real‑world retrospective study

**DOI:** 10.1007/s10238-023-01088-z

**Published:** 2023-06-08

**Authors:** Daniele Mengato, Maria Mazzitelli, Andrea Francavilla, Monica Bettio, Lolita Sasset, Nicolò Presa, Lisa Pivato, Sara Lo Menzo, Marco Trevenzoli, Francesca Venturini, Dario Gregori, Anna Maria Cattelan

**Affiliations:** 1 University of Padua, Padua, Italy; 2 Hospital Pharmacy Unit, Padova University Hospital, Padua, Italy; 3 Infectious and Tropical Diseases Unit, Padova University Hospital, Padua, Italy; 4 Department of Cardiac Thoracic Vascular Sciences and Public Health, University of Padua, Padua, Italy; 5 Department of Molecular Medicine, University of Padua, Padua, Italy

**Correction to: Clinical and Experimental Medicine** 10.1007/s10238-023-01036-x

The original version of this article unfortunately contained a mistake. Modifications have been made in the following section Abstract, Introduction, Methods, Outcomes, Statistical analysis, Results, Discussion and Conclusions.

The original article has been corrected.

